# Analysis of volatile metabolites from in vitro biofilms of *Pseudomonas aeruginosa* with thin-film microextraction by thermal desorption gas chromatography-mass spectrometry

**DOI:** 10.1007/s00216-020-02529-4

**Published:** 2020-03-20

**Authors:** Timo Koehler, Imke Ackermann, Dominik Brecht, Florian Uteschil, Jost Wingender, Ursula Telgheder, Oliver J. Schmitz

**Affiliations:** 1 Applied Analytical Chemistry, University of Duisburg-Essen, Universitaetsstr. 5, 45141 Essen, Germany; 2 Teaching and Research Center for Separation, University of Duisburg-Essen, Universitaetsstr. 5, 45141 Essen, Germany; 3 Aquatic Microbiology, Environmental Microbiology and Biotechnology, University of Duisburg-Essen, Universitaetsstr. 5, 45141 Essen, Germany; 4 Instrumental Analytical Chemistry, University of Duisburg-Essen, Universitaetsstr. 5, 45141 Essen, Germany

**Keywords:** *Pseudomonas aeruginosa*, Biofilm, Thin-film microextraction, Thermodesorption, TD-GC-qMS

## Abstract

Cystic fibrosis (CF) is an autosomal recessive inherited disease which leads to a production of thickened mucus in the airways. These conditions are conducive to poly-microbial infections, like chronic lung infection, in which *Pseudomonas aeruginosa* (*P. aeruginosa*) is the major pathogenic bacterium colonizing CF lungs at the end of the lifetime of CF patients. This in vitro study uses a *P. aeruginosa* biofilm model under partly cystic fibrosis conditions, with a sampling of volatile extracellular metabolites. The gas sampling was done with thin-film microextraction (TFME) and commercial polydimethylsiloxane (PDMS) films, whereas the analysis of loaded films was done by gas chromatography coupled to quadrupole mass spectrometry and thermodesorption (TD-GC-qMS). For this purpose, two commercially available films were characterized by means of thermogravimetry coupled to a qMS with atmospheric pressure photo ionization (TG-APPI-qMS), regarding homogeneity and temperature stability. The selected film was cleaned using a method developed in this study. The TD-GC-qMS method was successfully used for standards of volatile metabolites which were known to be produced by *P. aeruginosa*. Limits of detection and quantification of the method for middle and less polar compounds in low nanomolar range (0.5 nM and 1.5 nM) were achieved. The developed method was finally applied to investigate the extracellular volatile metabolites produced by biofilms of the strain *P. aeruginosa* DSM 50071 under aerobic and anaerobic conditions. In sum, eleven metabolites could be found under both conditions. Furthermore, it was shown in this study that different oxygen conditions (aerobic and anaerobic) resulted in emitting different extracellular volatile metabolites. Specific metabolites, like 1-undecene (aerobic) and 2-undecanone (anaerobic), could be identified. The results are promising, in that the biofilm model may be applicable for the identification of *P. aeruginosa* under clinical conditions. Furthermore, the model could be the basis for studying extracellular volatile metabolites from different mono- or co-cultures of various bacteria, as well as the implementation of pulmonary conditions, like these in CF lungs. This possibility allows the development of a non-invasive “at-bedside” breath analysis method for CF patients in focus of various bacterial infections.

**Electronic supplementary material:**

The online version of this article (10.1007/s00216-020-02529-4) contains supplementary material, which is available to authorized users.

**Figure:**
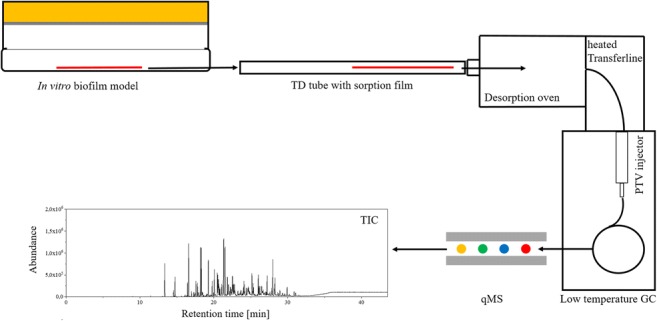
Graphical abstract

## Introduction

Cystic fibrosis (CF) is an autosomal recessive inherited disease that is caused by a dysfunction of the cystic fibrosis transmembrane conductance regulator (CFTR). This protein is a chloride ion transport channel that maintains osmotic balance across epithelia in the human body [1]. In CF lungs, CFTR dysfunction leads to electrolyte imbalance with the production and deposition of a thickened mucus obstructing the airways and serving as a nutrient source for bacteria. These conditions are conducive to poly-microbial colonization of the lungs, including diverse bacterial pathogens, which vary in their abundance during the different stages of the disease [1]. The gram-negative bacterium *Pseudomonas aeruginosa* (*P. aeruginosa*) is an opportunistic pathogen that represents a common causative agent of chronic lung infection resulting in a progressive decline of pulmonary function in CF patients [2, 3]. The persistence of *P. aeruginosa* in the airways is based on the formation of biofilms, which are bacterial aggregates embedded in a self-produced matrix of extracellular polymeric substances, including polysaccharides, proteins and deoxyribonucleic acid (DNA) [4]. *P. aeruginosa* is able to actively penetrate into the mucus, where hypoxic or anaerobic conditions prevail, and grows via anaerobic respiration with nitrate as the terminal electron acceptor (denitrification) [5, 6]. The biofilm mode of growth promotes chronic infections because biofilms show increased tolerance to antibiotics and protection from phagocytosis as well as from other innate and adaptive host immune defence mechanisms. The failure to effectively clear mucus from CF airways also contributes to the chronic status of the infection. Although *P. aeruginosa*–infected CF patients are usually treated with the aid of antibiotics, a chronic bacterial infection can hardly be prevented. An epidemiological link between respiratory tract infection with *P. aeruginosa* and morbidity and mortality rates in CF has been reported [2].

Early and accurate detection of *P. aeruginosa* infection is essential for optimized patient management and targeted antimicrobial treatment. *P. aeruginosa* lung infection is commonly diagnosed by culture of airway samples such as expectorated or induced sputum, oropharyngeal swabs and bronchoalveolar lavage [7]. However, there are limitations in the recovery of samples suitable for cultural detection of *P. aeruginosa*, since the procedure for obtaining specimens from patients may often be time consuming and invasive [7]. Less time-consuming and non-invasive tools are desirable for easier and more rapid diagnosis of *P. aeruginosa* infection that may enable more successful treatment and eradication of the bacteria before chronic infection is established. Molecular biomarkers of *P. aeruginosa* are useful for the identification of this organism in CF patient samples. Analysis of the extracellular volatile metabolome (volatilome), in particular detection of volatile organic compounds (VOCs), in exhaled breath has been proposed as an alternative diagnostic method for identifying pathogens such as *P. aeruginosa* on the basis of characteristic fingerprints [8, 9]. It is assumed that VOCs produced by pathogens in infected airways are exhaled and thus provide a potential for early non-invasive detection.

In a number of in vitro studies, VOCs have been analysed by headspace analysis of *P. aeruginosa* cultures under defined laboratory conditions. Based on these studies, acetic acid, acetaldehyde, acetone, 2-butanone, 2-nonanone, 1-undecene, 2,4-dimethyl-1-heptene, ethanol, 1-decanol, hydrogen sulphide, dimethyl sulphide, dimethyl disulphide, dimethyl trisulphide, methanethiol and hydrogen cyanide were identified as potential metabolites [10–15]. The disadvantage is the limited comparability between the studies due to different experimental conditions, e.g. the use of different strains (genomic variation), selection of different growth conditions (culture media, incubation time and temperature), use of bacteria from different growth phases, varying durations of headspace sampling, choice of VOC pre-concentration method and type of chemical analysis [8]. In volatilome studies, *P. aeruginosa* is usually grown in liquid media as planktonic cultures under aerobic conditions. However, as mentioned above, in CF lung infections, *P. aeruginosa* forms biofilms under hypoxic or anaerobic conditions. Based on these findings, in the current study, we have developed an in vitro biofilm system that mimics more closely growth conditions in CF airways. The model can be used under both aerobic and anaerobic conditions. In addition, it is universally applicable to study the volatile metabolome of various bacteria associated with CF.

Sampling of the volatile extracellular metabolites is done by thin-film microextraction (TFME), a membrane-based extraction and enrichment method based on solid-phase microextraction (SPME) [16]. This method was developed by Bruheim et al. [17] for the extraction and enrichment of polycyclic aromatic hydrocarbons from aqueous samples with subsequent analysis by a gas chromatograph coupled to mass spectrometer (GC-MS). The sorption phase consists of polydimethylsiloxanes (PDMS) or mixtures with PDMS [16]. Nowadays, TFME is used for the analysis of volatile organic compounds, like aromatics, herbicides, polycyclic aromatic hydrocarbons, pesticides, chlorobenzenes, less volatile hydrophobic compounds, sebum, explosives, illegal drugs and benzodiazepens [16], whereby the analytes are adsorbed from different, partly highly complex matrices. For example, the TFME is used in food samples [18], water, fuel, fish tissue, soil, sediment, human skin, urine and blood [16]. The advantage of TFME over comparable and more well-established processes such as SPME and stir bar sorptive extraction (SBSE) is the greater surface-to-volume ratio of the extraction phase, which results in faster extraction rates and consequently in increasing speed of equilibrium. Furthermore, the volume of the extraction phase is significantly larger compared with SPME, which results in a higher sensitivity [16]. TFME can be applied to solid, liquid or gaseous samples in either immersive or headspace mode. In the case of liquid samples, the thin film is immersed in the liquid or the sampling can be performed in the headspace of the liquid. The headspace mode is operated by sampling the gaseous analytes in the gas space above a liquid or solid sample [18]. The analysis of volatile organic compounds is usually carried out by GC-MS. The transfer of the analytes, adsorbed by the thin film, can be carried out directly by a thermal desorption unit, or solvent assisted. Jiang and Pawliszyn explained the advantages and disadvantages of direct and solvent-assisted desorption [16]. Direct desorption has the advantage, in that all adsorbed analytes can be transferred into the analytical system because low abundant analytes can also be detected. Because of the simple and commercially available possibility of coupling thermodesorption (TD) with GC-MS with a programmed temperature vaporizer (PTV) injector, this is the most commonly used combination when using TFME [16].

Because of the lack of a comparable method, we have carried out a detailed method development for sampling and analysis of VOCs. Therefore, the stability of TFME material is investigated by means of thermogravimetry in combination with mass spectrometry. In addition, a cleaning and conditioning procedure was developed. As mentioned above, VOCs emitted from *P. aeruginosa* show a broad spectrum of polarity. Therefore, an analytical system including a GC with a cooled oven system, a PTV injector and a quadrupole mass spectrometer (qMS) was used. After validation of the method, the complete system was used to investigate the influence of different growth conditions (aerobic and anaerobic) on the metabolic fingerprint of *P. aeruginosa*.

## Material and methods

### Chemicals

The TD-GC-qMS method development was done using dimethyl sulphide (**1**, anhydrous, ≥ 99%), 2-methylbutanal (**2**, 95%), dimethyl disulphide (**3**, ≥ 98%), 2-hexanone (**4**, analytical standard), 1-octanol (**6**, anhydrous, ≥ 99%), 2-nonanone (**7**, ≥ 99%), 1-undecene (**8**, 97%), 1-decanol (**9**, ≥ 9 8%) and 2-aminoacetophenone (**10**, analytical standard) purchased from Sigma-Aldrich (Taufkirchen, Germany), and 2-heptanone (**5**, ≥ 98%) purchased from Merck KGaA (Darmstadt, Germany).

To verify the analysis of cyclic siloxanes by atmospheric pressure ionization (APPI), coupled with thermogravimetry (TG) and a qMS, three cyclic siloxanes were analysed as single standards. Octamethylcyclotetrasiloxane (D4, 98%, Alfa Aesar, Karlsruhe, Germany), decamethylcyclopentasiloxane (D5, 97%, Alfa Aesar, Karlsruhe, Germany) and dodecamethylcyclohexasiloxane (D6, 95%, Alfa Aesar, Karlsruhe, Germany) were used.

For all purposes, liquid chromatography-mass spectrometry (LC-MS) grade methanol from VWR (Leuven, Belgium) and ultrapure water, generated with a water purification system from Sartorius Stedim (Göttingen, Germany), were used. For cleaning purpose, Decon 90 from VWR (Leuven, Belgium) was used.

### Sample preparation

#### Liquid samples

For the development of the TD-GC-qMS method, the above-mentioned standards were prepared as single standards in methanol with each 100 mM. Afterwards, a multi-standard was prepared with a concentration of 5 mM for each single standard. In order to determine the limit of detection and limit of quantification, the 3*σ* method [19] was used and the multi-standard was diluted with water to concentrations between 5 fM and 500 μM. To determine the LOD and LOQ, cutted PDMS films from manufacturer A (0.35 mm × 27.5 mm × 0.45 mm; Goodfellow GmbH, Hamburg, Germany) were used. The PDMS films are half the size of the films used in the in vitro model. The films were loaded using the immersive method from the aqueous solutions of the multi-standard (*c* = 5 fM–500 μM). To load the films, a 2-mL crimp vial (CS Chromatographie Service, Langerwehe, Germany) was filled with 2 mL of the multi-standard. A sorption time of 2 h was chosen. Sorption was also carried out without stirring and at room temperature (approx. 23 °C).

#### Biofilm model

**Fig. 1:**
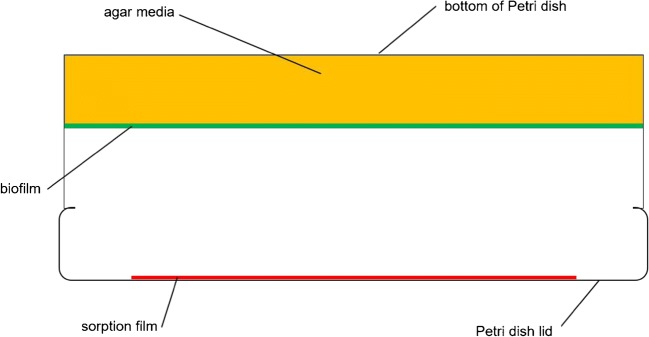
Schematic of the in vitro biofilm model

Biofilms were cultivated as confluent bacterial lawns on the surface of agar media as a simple in vitro biofilm model of *P. aeruginosa* (Fig. 1).

Type strain *P. aeruginosa* DSM 50071 (ATCC 10145) was used for biofilm cultivation. Growth media were LB agar (Lennox) containing (per L) 10 g tryptone, 5 g yeast extract, 5 g NaCl and 15 g agar, with a final pH value of 7.0 ± 0.2 (Carl Roth GmbH + Co. KG, Karlsruhe, Germany) and LB agar supplemented with KNO_3_ (final conc. 100 mM; Merck KGaA, Darmstadt, Germany). The agar media (20 mL) were filled into glass Petri dishes (diameter, 90 mm). A PDMS film strip (0.35 mm × 55 mm) was placed in the centre of the lids (Fig. 1). Pre-cultures of *P. aeruginosa* were grown on LB agar plates at 37 °C overnight. Single colonies of these cultures were suspended in 0.14 M NaCl solution; the cell density was determined with a Thoma counting chamber and the suspension was adjusted to a concentration of 10^8^ cells mL^−1^. Aliquots of 0.1 mL bacterial suspension were spread-plated on the surface of the agar media. LB agar plates were incubated aerobically and LB agar plates with added potassium nitrate were incubated anaerobically at 37 °C for 48 h. Anaerobic cultivation was carried out in anaerobic jars with an oxygen-free atmosphere generated by Anaerocult® A (Merck KGaA, Darmstadt, Germany). Indicator strips (Anaerotest®, Merck KGaA, Darmstadt, Germany) were used to confirm the generation of anaerobic conditions inside the jars. All plates were incubated upside down (Fig. 1). Volatile compounds released during cultivation were trapped using TFME.

#### Thin-film microextraction

PDMS was used as sorption material for TFME and two commercial PDMS films were tested using our developed TG-APPI-qMS system [20]. One PDMS film was purchased from Goodfellow GmbH (manufacturer A, Hamburg, Germany) with a film thickness of 0.45 mm (± 10%). The second PDMS film was purchased from a manufacturer in the USA (manufacturer B, Interstate Specialty Products, Sutton, MA, USA) with a film thickness of 0.36 mm (± 0.05 mm). The film of manufacturer A was cleaned before analysis using the following procedure. A two-step solvent cleaning was done. In the first step, the films were placed in LC-MS grade methanol and shaken on a vortex mixer (SM 25, Edmund Bühler GmbH, Bodelshausen, Germany) for 90 min. Five percent Decon 90 in water was used as a second solvent cleaning step and the films were also shaken on the vortex mixer for 90 min. Decon 90 is a surface-active cleaning agent which is biodegradable, phosphate-free and bactericidal. After this step, the films were rinsed with ultrapure water and transferred to the thermal cleaning device. The Gerstel Tube Conditioner 2 (TC 2, Gerstel GmbH & Co. KG, Mülheim an der Ruhr, Germany) was used for thermal cleaning of the films. The films were put in the TDS tubes and ten of them were placed into the TC 2. The optimized method includes 6 cycles of a temperature programme from 50 to 200 °C (hold 120 min) with a rate of 10 °C min^−1^ and a nitrogen flow of 24 mL min^−1^. For storage, the films were placed in a 20-mL vial (Macherey-Nagel, Dueren, Germany) filled with argon (ARCAL Prime, 99.998%, Air Liquide, Düsseldorf, Germany). The films were thermally treated at 200 °C for 1 h by TC 2 before use.

### Instrumentation

#### TD-GC-qMS

Cleaned TFME films were analysed with a thermodesorption system (TDS) directly coupled to GC-MS. The system is set up of a Gerstel TDS-A2 autosampler, a Gerstel TDS 3, the Gerstel cold injection system (CIS 4, Gerstel GmbH & Co. KG, Mülheim an der Ruhr, Germany), an Agilent 6890 Gas chromatograph and an Agilent 5975 MSD Mass spectrometer (Agilent Technologies Inc., Waldbronn, Germany). Adsorbed analytes were thermally desorbed in the TD system at 200 °C using a desorption flow of 60 mL min^−1^ helium (ALPHAGAZ 1, 99.9%, Air Liquide, Düsseldorf, Germany). The TDS is connected to the cold injection system CIS 4 using a metal transfer line with a temperature of 300 °C. In the CIS 4, the analytes were trapped on deactivated glass wool at a temperature of − 10 °C. The liner was subsequently heated up to 270 °C with a temperature programming rate of 12 °C s^−1^ and transferred into the GC column. To refocus the analytes at the head of the column, the oven temperature firstly was held at − 10 °C using liquid nitrogen. The chromatographic separation was performed on a DB-1 column (Agilent Technologies Inc., Waldbronn, Germany) with a length of 30 m and an inner diameter of 0.25 mm and a film thickness of 1 μm using a helium flow (ALPHAGAZ 1, 99.9%, Air Liquid, Düsseldorf, Germany) of 1 mL min^−1^. The oven temperature was increased from  −10 to 325 °C with a temperature programming rate of 10 °C min^−1^. GC and MS were connected with a heated transfer line at a temperature of 280 °C. The MS was used with electron impact ionization at 70 eV, with a scan range of m/z 40–600 Da and a scanning rate of 2.24 scans s^−1^.

The identification of the analytes from the obtained electron impact MS (EI-MS) spectra was performed using the NIST database (version: NIST17; 2017; National Institute of Standards and Technology).

#### TG-APPI-qMS

For thermal analysis of the commercial films, 5 mg of the films was weighed into a ceramic crucible. Each film was analysed three times, where different areas of the film were investigated. The samples were heated up from 30 to 900 °C with a temperature rate of 10 °C min^−1^ in the thermogravimetry STA 7200 from Hitachi High-Technologies (Chiyoda, Tokyo, Japan). Eleven millilitres per minute of the total 200 mL min^−1^ nitrogen flow was transferred to the quadrupole MS and 189 mL min^−1^ was transferred to the split exit of the system. The analytes were detected by a modified qMS (Chrommaster 5610, Hitachi High-Technologies, Chiyoda, Tokyo, Japan). Briefly, the mass spectrometer is equipped with a closed ion source chamber that holds a vacuum ultraviolet (VUV) Krypton lamp which emits photons at 117 and 124 nm (PKR 106, Heraeus, Hanau, Germany). The analytes are introduced via a gas-tight inlet port. Analyte ion transfer to the mass analyser is enhanced by an additional gas flow that was set to 450 mL min^−1^ by a mass flow controller (Bronkhorst High-Tech B.V., Ruurlo, Netherlands). The atmospheric pressure interface lenses (AP1 and AP2) were set to 40 and 20 V, respectively. The scan range was set to 50–700 Da and the dwell time to 2000 ms. A detailed description of the instrumental coupling of the thermogravimetry and the atmospheric pressure photo ionization quadrupole mass spectrometer (TG-APPI-qMS) is described in the literature [20]. To verify the ionization of cyclic siloxanes with atmospheric pressure ionization (APPI), D4–D6 standards were analysed. Therefore, 1 μL of the pure standard was weighed into an aluminium crucible. The TG was subsequently heated up from 50 to 500 °C with a temperature rate of 150 °C min^−1^ and an AP1:AP2 voltage of 90 V:10 V. In all mentioned TG-APPI-qMS analyses, the temperatures of the AP1, transferline and ion source were 120 °C, 325 °C and 250 °C, respectively.

## Results and discussion

### Selection of sorbent material

The TFME method was selected to analyse volatile metabolites in the headspace above the biofilms of *P. aeruginosa*. The selected model substances, based on the publication by Bos et al. [8], have different chemical and physical properties, e.g. polarity and vapour pressure. Besides the possibility of sorption of the analytes of interest on the sorbent, the criteria for the selection of the sorption material are the temperature stability for the desorption and the uniform production of the films to guarantee a reproducible analysis. PDMS was selected as the sorbent material because of the higher temperature stability and the sorption suitability of analytes with a higher vapour pressure range in comparison with, e.g., polyoxymethylene [21, 22]. Sprunger et al. [23] have calculated the sorption coefficients of PDMS and analytes in the gas phase. These findings indicate that PDMS is the best sorption phase for this approach, as a broad spectrum of diverse volatile metabolites of *P. aeruginosa* is expected [8, 23]. Commercial PDMS films were selected to ensure large-scale and reproducible production. On basis of these preliminary considerations, the PDMS films of two manufacturers were selected. Another analytically relevant parameter of the films is the film thickness. Because of the large gas volume above the biofilm, a film thickness of about 0.4 mm was selected. A film with a high capacity is necessary to ensure a reproducible sampling. Based on the available products of the two manufacturers, the films described in the “Thin-film microextraction” section were selected.

### Characterization of sorbent material by TG-APPI-qMS

The selected commercially available PDMS films were characterized by TG coupled to a qMS with an APPI source, prior to the cleaning process. The investigation of PDMS and blends with PDMS by means of TG has been previously reported by Nair et al. [24]. It was shown that the formation of cyclic siloxanes with different ring sizes is induced by temperature and depends on the additives of the PDMS mixture [24]. The characterization of PDMS film material from two manufacturers was firstly performed by thermal analysis. Triplicates of the derivative thermogravimetry (DTG) curves, normalized to the initial weight, are shown in Fig. S1 (see Electronic Supplementary Material, ESM). Comparing the DTG curves, it maybe be stated that the decomposition of the films from manufacturer B starts to occur at 100 °C. In contrast, the films of manufacturer A decompose starting from a temperature of 300 °C. Considering the white, opaque appearance of films from manufacturer A and taking into account the results from Nair et al., manufacturer A may use additives such as titanium dioxide for the production of the PDMS films [24]. In addition to higher temperature stability, the PDMS film from manufacturer A also displays a higher reproducibility [24]. This was investigated using the percentage standard deviation of the value at the apex of the DTG curves. For the three replicates of the films from manufacturer A, with a maximum at 620 °C, a percentage standard deviation of 1.7% has been calculated. The films from manufacturer B, with a maximum at 680 °C, show a 14.5-fold higher standard deviation with 24.7%. If the temperature at the peak apex is compared, it could be assumed that the films of the manufacturer B are more temperature stable. However, it can also be observed that the loss of mass with the microbalance can already be detected at temperatures above 100 °C for the film of manufacturer B. The working range of the PDMS films in the application described in this publication is due to the wide range of polarity of the VOCs at 200 °C. Furthermore, at a temperature of 200 °C according to manufacturer A, the bleeding of the films is the lowest, so that this temperature is indicated as the upper working temperature. The mass loss in the relevant temperature range from manufacturer A is 6 ± 0 μg, while in the same temperature range from manufacturer B, a mass loss of 26 ± 5.6 μg can be observed. Less contamination from the films leads to a lower background and, therefore, better accuracy and lower detection limits for the TD-GC-qMS analysis are expected. Nevertheless, higher temperature stability coupled with minimal elution of substances is beneficial for TFME in combination with thermal desorption. Cyclic siloxanes are expected to be the main impurity eluting from the PDMS films. This assumption is based on the results of Nair et al. and the knowledge about the thermal decomposition of PDMS and was confirmed with the suspect target approach, using the TG-APPI-qMS analytical platform. To verify whether cyclic siloxanes can be ionized with an APPI source, the cyclic siloxanes D4, D5, and D6 were analysed as analytical standards directly with the TG-APPI-qMS (see ESM Fig. S2). The mass traces of D4, D5, and D6 show highest intensities for [M-CH_3_]^+^ as a function of time (ESM Fig. S3). Based on these findings, TG-APPI-qMS analysis of the PDMS films was performed. Fig. S4 (see ESM) describes the EIC of the cyclic siloxanes from D3 to D7 and the total ion chromatogram (TIC). Analogous to the DTG curves, the TICs show a later elution of the substances from the film of manufacturer B. With the help of the TICs and EICs, it can be shown that mainly cyclic siloxanes with a silicon atom number of three to seven elute from the films. Furthermore, it can be observed that all considered cyclic siloxanes (D3 to D7) in the films of manufacturer A elute homogeneously and in a Gaussian peak. On the other hand, in the case of manufacturer B, elution of the cyclic siloxane D5 takes place as early as 10 min. This corresponds to a temperature of 126 °C. This is followed by a two-stage release of D4 to D7. The cyclic siloxane D3 is less released by both manufacturers. The observed higher temperature stability and the homogeneous release of the cyclic siloxanes can be, as already discussed by Nair et al., attributed to a stabilization of the PDMS by additives [24]. Indeed, due to limited information about the production process, these interpretations are based on educated guesses. Furthermore, it can be observed that the thermal decomposition of the films from manufacturer B takes places in at least four steps (ESM Fig. S4). The decomposition steps 1 to 3 results from cyclic siloxanes, whereas in the last decomposition step, another compound with a m/z of 353 elutes (see ESM Fig. S5). The characterization of the films by TG and TG-APPI-qMS showed that the film of manufacturer A was manufactured more homogeneously and temperature stable compared with the second investigated manufacturer B. Based on these two findings, the film of manufacturer A was selected for the TFME approach. Furthermore, the results demonstrate that TG-APPI-qMS has a promising potential for quality control in the future.

### Cleaning method for sorption film and analytical TD-GC-qMS method

**Fig. 2:**
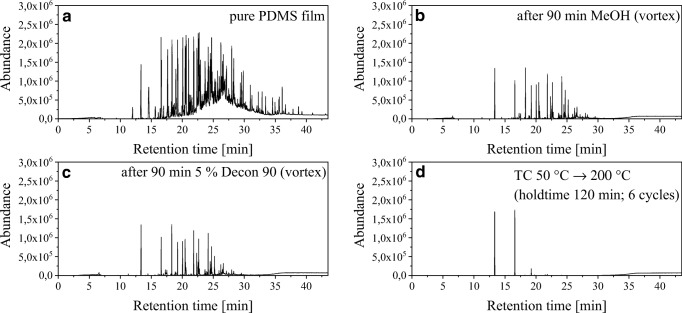
TICs from the TFME films **a** after delivery and without purification, **b** after 90 min in methanol, **c** after 90 min in methanol and 5% Decon 90 and **d** after the entire purification procedure including the thermal cleaning. The films were analysed with a TD-GC-qMS

The direct use of PDMS films is desirable. However, a conditioning step is inevitable because of contamination, which is presumed to originate from the production process of the films. The chromatogram in Fig. 2a shows a total ion chromatogram of a film that was desorbed and analysed directly after delivery with the TD-GC-qMS. A two-stage cleaning procedure consisting of a cleaning step with solvents and a thermal cleaning step was carried out based on work from Riazanskaia et al. [25]. The cleaning procedure has been optimized regarding washing times and times of thermal treatment (results not shown). The cleaning processes were investigated stepwise. Figure 2 shows the result of each individual purification step by TD-GC-qMS.

It can be seen from Fig. 2b that the purification with methanol results in a significant decrease of the contaminations. However, satisfactory purification with methanol was not achieved. After further purification with 5% Decon 90 solution (Fig. 2c), more contaminations have been removed, such as observed in the region between 25 and 30 min. Indeed, there are a lot of contaminations visible in the retention time region between 15 and 30 min. Based on the assumption that the contaminants are substances with a low to medium boiling range, thermal cleaning was selected. As a result of the entire cleaning process, the sorption material shows a significant decrease of the contaminations. Further removal of the remaining contaminations, observed in Fig. 2d, is not possible on a methodological basis. The three peaks can be assigned to be the cyclic siloxanes D3, D4, and D5. They are originated from the PDMS film and describe the background of the used material and hence cannot be prevented or removed thereby.

**Fig. 3:**
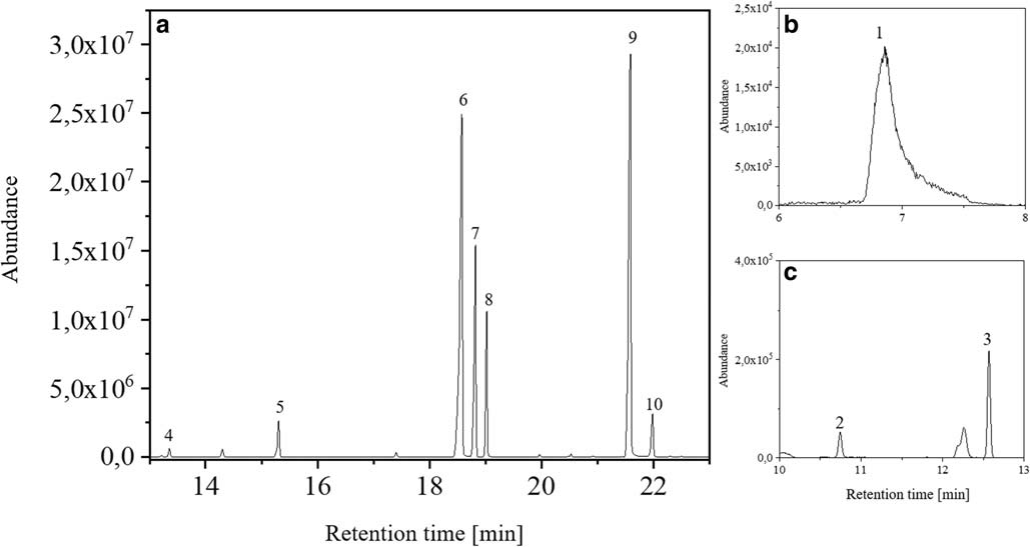
Result of the immersive method sorbed model substances on the TFME film. The TIC in the retention time range of **a** 13–23 min, **b** 6–7.5 min and **c** 10–13 min is shown. The identified model substances are dimethyl sulphide (1, rt = 6.86 min), 2-methylbutanal (2, rt = 10.75 min), dimethyl disulphide (3, rt = 12.57 min), 2-hexanone (4, rt = 13.35 min), 2-heptanone (5, rt = 15.31 min), 1-octanol (6, rt = 18.58 min), 2-nonanone (7, rt = 18.82 min), 1-undecene (8, rt = 19.02 min), 1-decanol (9, rt = 21.60 min) and 2-aminoacetophenone (10, rt = 22.00 min)

The TD-GC-qMS method used for gas-phase metabolome analysis was developed for the volatile metabolites produced by *P. aeruginosa*. The diversity of the metabolites produced by *P. aeruginosa* demonstrates the challenge of developing a method that covers a broad range of metabolites. Bos et al. [8] reported several metabolites originating from *P. aeruginosa*, namely dimethyl sulphide and 1-undecene, among others. The example of these two metabolites demonstrates the challenge of developing a method for the metabolome analysis of *P. aeruginosa*. Dimethyl sulphide has a boiling point of 37 °C, whereas 1-undecene has comparably higher boiling point of 192–193 °C. Furthermore, metabolites of a broad range of polarity were already published as metabolites originating from *P. aeruginosa* [8]. Therefore, a cryo focusing of the thermodesorbed analytes on the liner at − 10 °C was used in combination with oven cooling to refocus the analytes on the column head after transfer from the liner onto the column. With this method, analytes with a very low-boiling point can be analysed, such as dimethyl sulphide. Furthermore, a decrease of the peak widths to about 3 s could be achieved (see ESM Fig. S6 and Table S1). Figure 3 shows the resulting chromatogram of a mix standard of ten potential volatile metabolites of *P. aeruginosa* with a TFME from 50 μmol L^−1^ solution using the developed TD-GC-qMS method.

Figure 3 demonstrates that analytes with a high vapour pressure (dimethyl sulphide (**1**) and 2-methylbutanal (**2**)) can be detected with TD-GC-qMS. Furthermore, it is observed that Gaussian peaks can be obtained, except for dimethyl sulphide. Furthermore, 2-nonanone (**7**) can separate from 1-undecence (**8**). This has already been described as a critical separation problem by Zscheppank et al. [26]. In addition, 1-octanol (**6**) can also be baseline-separated from 2-nonanone (**7**). All chosen metabolites are successfully detected as Gaussian peaks with the developed analytical method, with exception of dimethyl sulphide.

### Determination of LOD and LOQ

For a more accurate characterization of the TD-GC-qMS method, the method detection and quantification limits were determined. The standards listed in Table 1 were prepared and analysed with TFME in immersive mode. The concentration of the individual analytes in the multi-standard was varied from 5 fM to 500 μM. Subsequently, the analysis of the films was carried out with the described TD-GC-qMS method. The determination of LOD and LOQ was carried out by using the TICs according to the 3*σ* method of Kaiser and Specker [19]. With the developed method, LODs of 500 fM and LOQs of 1.5 nM can be achieved for medium- and high-boiling analytes. Low-boiling components such as 2-methylbutanal and dimethyl disulphide have a LOD of 150 nM and a LOQ of 500 nM. Very low–boiling analytes such as dimethyl sulphide have very high detection and quantification limits compared with the other analytes with a LOD of 150 μM and a LOQ of 500 μM. All calculated values for LOD and LOQ are shown in Table 1.

**Table 1:** LOD and LOQ of ten published possible metabolites of *P. aeruginosa* with TD-GC-qMS in nanomolar concentration, as well as the corresponding retention time in minutes

Substance	Retention time (min)	LOD (nM)	LOQ (nM)
Dimethyl sulphide (**1**)	6.86	150,000	500,000
2-Methylbutanal (**2**)	10.75	150	500
Dimethyl disulphide (**3**)	12.57	150	500
2-Hexanone (**4**)	13.35	5	15
2-Heptanone (**5**)	15.31	0.5	1.5
1-Octanol (**6**)	18.58	0.5	1.5
2-Nonanone (**7**)	18.82	0.5	1.5
1-Undecene (**8**)	19.02	0.5	1.5
1-Decanol (**9**)	21.60	0.5	1.5
2-Aminoacetophenone (**10**)	22.00	0.5	1.5

The substances listed in Table 1 were selected on the basis of a literature search on extracellular metabolites of *P. aeruginosa.* To test the general use of our method, the LODs were determined without matrix. Therefore, these values are of course only approximate, since neither the nutrient medium nor the biofilm was considered as matrix. But even if we would spike the biofilm with stable isotopic compounds of these analytes, these LODs would be only approximations, because the conditions in the breath air are completely different.

### Validation of in vitro biofilm model using TD-GC-qMS

The in vitro biofilm model was checked with the multi-standard used for method development. For this purpose, 100 μL of the multi-standard with a concentration of 500 μM for each model substance was inoculated onto the nutrient medium of the biofilm model. The test was carried out for both aerobic and anaerobic conditions with LB-Lennox and potassium nitrate–supplemented LB-Lennox medium, respectively. The chromatograms obtained, including the assignment of the model substances by means of the NIST database, are shown in Fig. S7 (see ESM). The use of the in vitro biofilm model was successful in both conditions for 8 out of 10 analytes. However, the absence of two analytes, dimethyl sulphide and dimethyl disulphide, was observed. The lack of detection of dimethyl sulphide and dimethyl disulphide can be associated with the vapour pressure [27, 28]. It is assumed that these analytes already evaporate during the plating of the standard solution on the nutrient medium and get into the gas chamber before the introduction of the TFME film and thus elude detection. In comparison with the cleaned films, a high background can be detected. The main background results from the agar medium (see ESM Fig. S8).

### Investigation of metabolites using in vitro biofilm model

**Fig. 4:**
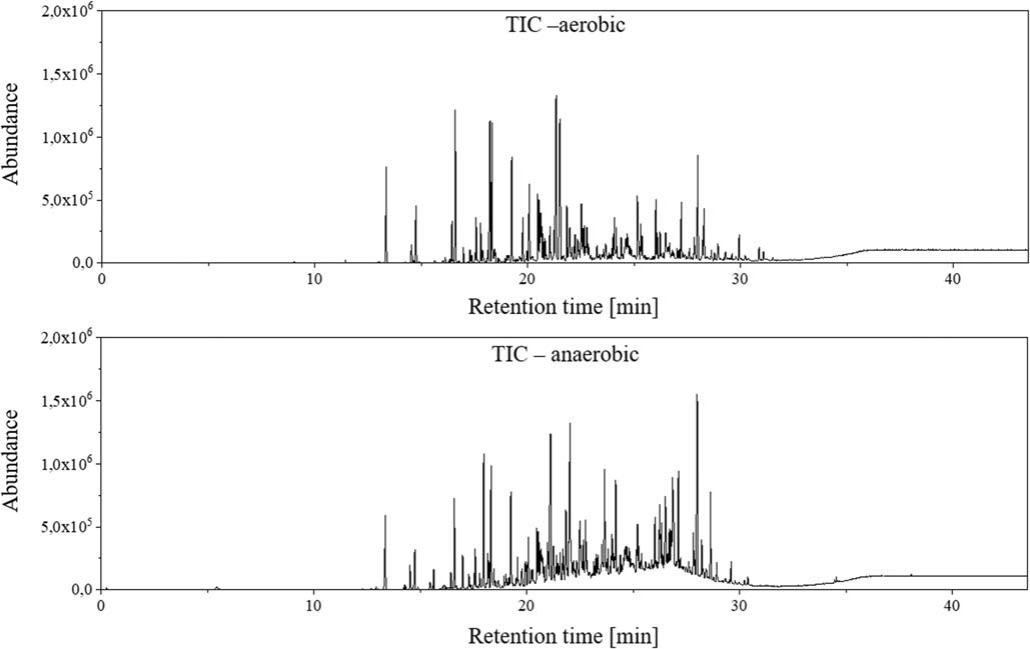
TIC of the analyses with the biofilm model using strain *P. aeruginosa* DSM 50071 under aerobic (top chromatogram) or anaerobic conditions (bottom chromatogram)

After analysis of the multi-standard in the agar medium, the volatile metabolites of the bacterial strain *P. aeruginosa* DSM 50071 were investigated. Both conditions (aerobic and anaerobic) were examined as before. The TICs of these two analyses are shown in Fig. 4. A total of nine biological replicas were analysed in aerobic and anaerobic conditions and the TICs are shown in ESM Figs. S9 and S10, respectively.

Figure 4 demonstrates that significantly more analytes are detected under anaerobic conditions. However, there is no baseline separation possible with a one-dimensional GC due to peak overlaps and coelution observed in the chromatogram between 20 and 30 min. Nevertheless, it is possible to identify several analytes using NIST database. All chromatograms were processed by a blank subtraction. With these pre-processed data, an assessment was carried out with regard to selective metabolites that occurred exclusively under one of the two conditions considered. Taking these conditions into account, eight metabolites, namely acetic acid (**11**), 2-methyl-quinoxaline (**12**), 1-undecene (**8**), decyloxirane (**13**), methylpyrazine (**14**), cyclododecane (**15**), propanoic acid (**16**) and butanoic acid (**17**), were detected which were observed only under aerobic conditions. Three metabolites, namely 2-undecanone (**18**), 2-nonanone (**7**) and 1-methoxy-2-propanone (**19**), were identified that could only be observed under anaerobic conditions. The retention time (SD less than 5 s) and the NIST score (greater than 80%) were used as criteria for identification. Table 2 lists the identified metabolites. The identification was performed using two independent electron impact MS databases. First, the NIST database was searched for hits with a score higher than 80%. After that, a metabolite-specific GC-MS database called MassBank of North America (MONA) by Oliver Fiehn was applied to the dataset [29]. Only hits with a score over 80% in both libraries were declared as identified. The second database was used because of its specificity to metabolites. In general, the results obtained with the MONA database were close to those from the commonly used and larger NIST database. Already six of the metabolites identified in this study have been described as volatile compounds of *P. aeruginosa* in previous publications. These are the metabolites acetic acid, 1-undecene, methylpyrazine, propanoic acid, 2-undecanone and 2-nonanone [11, 30–33]. However, five metabolites were identified, which, to our knowledge, have not yet been published as being present in the headspace of *P. aeruginosa* cultures, 2-methyl-quinoxalines, decyloxirane, cyclododecane, butanoic acid and 1-methoxy-2-propanone.

**Table 2:** Overview of the identified metabolites using the biofilm model for the strain *P. aeruginosa* DSM 50071. Aerobic and anaerobic growth conditions were chosen. The NIST and MONA [29] scores were shown as an average value of nine biological replicates with the related standard deviation

Metabolite	Ø RT (min)	Ø NIST score (%)	Ø MONA score (%) [31]
Aerobic conditions			
Acetic acid (**11**)	8.94 ± 0.07	97 ± 1	93 ± 1
2-Methyl-quinoxaline (**12**)	21.31 ± 0.01	95 ± 1	82 ± 1
1-Undecene (**8**)	18.21 ± 0.01	94 ± 1	93 ± 1
Decyloxirane (**13**)	21.49 ± 0.01	93 ± 1	90 ± 9
Methylpyrazine (**14**)	12.98 ± 0.01	90 ± 2	91 ± 4
Cyclododecane (**15**)	19.76 ± 0.01	87 ± 2	91 ± 2
Propanoic acid (**16**)	10.63 ± 0.03	86 ± 5	89 ± 11
Butanoic acid (**17**)	12.35 ± 0.03	80 ± 5	88 ± 4
Anaerobic conditions			
2-Undecanone (**18**)	21.09 ± 0.01	96 ± 1	88 ± 3
2-Nonanone (**7**)	17.95 ± 0.01	94 ± 1	85 ± 3
1-Methoxy-2-propanone (**19**)	5.91 ± 0.02	90 ± 1	85 ± 3

**Fig. 5:**
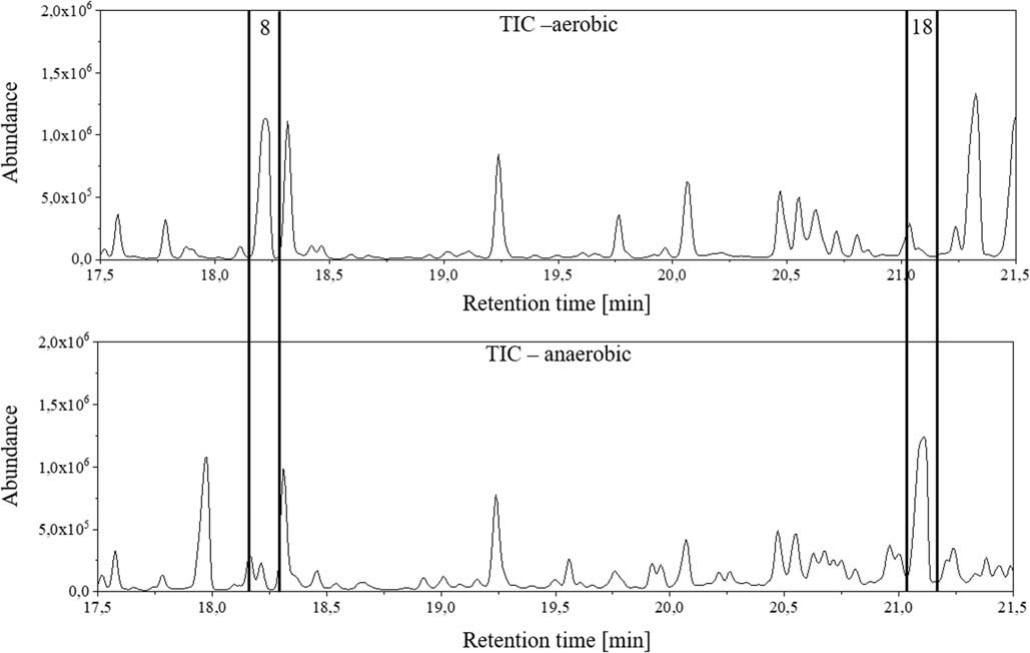
Example of the differentiation of growing conditions based on extracellular volatile metabolites. For this purpose, a section of the TICs shown in Fig. 4 is shown from 17.5 to 21.5 min for aerobic (top) and anaerobic (bottom) conditions. The numeral 8 denotes the metabolite 1-undecene, which is formed only under aerobic conditions. In the chromatogram of the anaerobic growth conditions, a peak at the same retention time can be observed. However, this peak results from the LBN nutrient medium and can be observed in the procedural blank, too. The second example (peak number 18) shows the 2-undecanone, which can only be detected under anaerobic conditions

In addition to the identification, a differentiation based on the identified metabolites was possible. To compare the two models regarding differences in metabolic products, the total ion chromatograms were closely investigated in the retention time range from 17.5 to 21.5 min. Figure 5 shows two examples of metabolites formed solely under one of the two conditions.

In Fig. 5, two metabolites are shown which are formed exclusively under one of the two conditions. 1-undecene (**8**) is formed only under aerobic conditions, whereas 2-undecanone (**18**) is formed only under anaerobic conditions. To assess the repeatability of the method, including biological variability, nine biological replicates were studied on two different days. The retention time, the area and the NIST database score between the biological replicates were compared. 1-undecene elutes at a retention time of 18.21 min ± 0.01 min. Furthermore, the area of 1-undecene has a relative standard deviation of 7.53%. This is a very small deviation in view of the high biological variability. The NIST score averages 94% with a standard deviation of 1%. Analogously, the same parameters for the 2-undecanone were calculated. This results in a retention time of 21.09 min ± 0.01 min and a relative standard deviation of the area of 9.44%. The NIST database score is 96% for 2-undecanone with a deviation of 1%. In addition, the variance in peak heights of the two metabolites was examined. Analysis of the nine biological replicates revealed a difference in heights of 13.2% and 12.4% for the 1-undecene and 2-undecanone, respectively. Considering the biological variability, the system deviations by 13% of the heights is highly acceptable.

## Conclusion

In this study, an in vitro model to analyse the extracellular volatile metabolites formed under biofilm conditions of *P. aeruginosa* was developed. The model could be the basis for studying extracellular volatile metabolites from various mono- and co-cultures under pulmonary conditions, like these in CF lungs. A methodology for sampling of extracellular volatile metabolites, using TFME, was developed and applied to the in vitro biofilm model. Thermal analysis helped to investigate the selected sorbent material by means of quality control. Furthermore, it could be shown that the ionization of cyclic siloxanes with APPI is possible in a TG-qMS system. The developed in vitro model was successfully validated using standards and real bacterial biofilms. The analysis of model metabolites was used to determine the LOD and LOQ in low nanomolar ranges. Eleven metabolites, for strain *P. aeruginosa* DSM 50071, were found with the developed methodology and it could be shown that differentiation between aerobic and anaerobic growing conditions based on volatile metabolites is possible.

Prospectively, the developed methodology, including in vitro model, sampling and analytical system, should be used for a comparison of well-characterized clinical isolates from *P. aeruginosa*, like strains PAO1 and FRD1, as well as fresh clinical isolates. The focus is on the identification of specific metabolites of mucoid strains, which mainly grow in CF lungs. Furthermore, the in vitro model should be improved in perspective of CF lung infection using artificial sputum medium, real sputum medium and multiple bacteria suspensions, to imitate the lung of CF patient in different stages of the disease. With this method, maybe volatile metabolites of *P. aeruginosa* and hopefully other bacteria could be determined as biomarkers. These biomarkers may then be detected by a non-invasive “at-bedside” breath target analysis method to detect severe lung infections with *P. aeruginosa* of CF patients at an early stage.

## Electronic supplementary material

ESM 1 (PDF 788 kb)
