# Supplementary material for: Analysis of volatile metabolites from in vitro biofilms of Pseudomonas aeruginosa with thin-film microextraction by thermal desorption gas chromatography-mass spectrometry
Source: Anal Bioanal Chem. 2020 Mar 20;412(12):2881–92. doi: 10.1007/s00216-020-02529-4 (PMC7196090; doi:10.1007/s00216-020-02529-4)
Supplement: Supplementary file 1 — (PDF 788 kb) [file 216_2020_2529_MOESM1_ESM.pdf]

Analytical and Bioanalytical Chemistry

Electronic Supplementary Material

Analysis of volatile metabolites from in vitro biofilms of *Pseudomonas aeruginosa* with thin-film microextraction by thermal desorption gas chromatography-mass spectrometry

Timo Koehler, Imke Ackermann, Dominik Brecht, Florian Uteschil, Jost Wingender, Ursula Telgheder, Oliver J. Schmitz

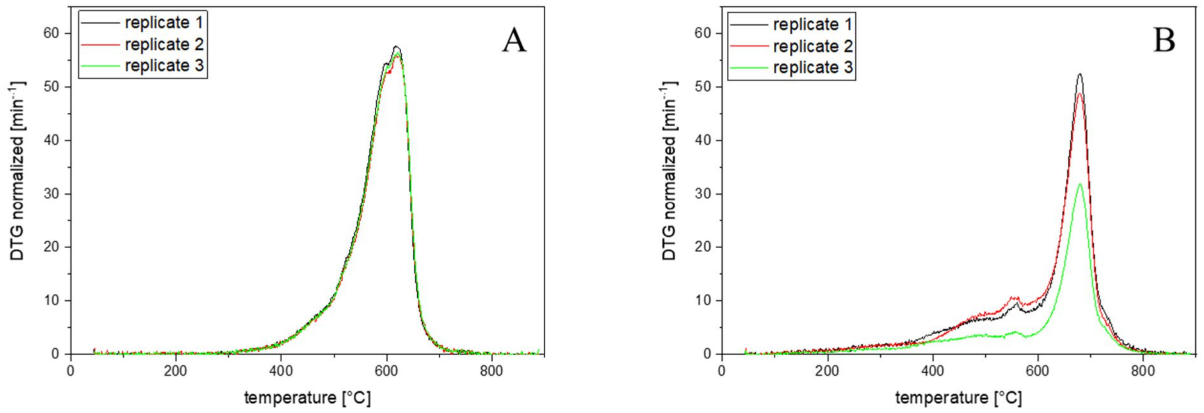

Fig. S1 DTG curves, normalized to the initial weight, for all three replicates (repl. 1 = black; repl. 2 = red; repl. 3 = green) of the PDMS films from two manufacturers (A and B). About 5 mg of the chosen film was weighted into an Al_2O_3 crucible and were heated up from 30 °C to 900 °C with a gradient of 10 °·min^{-1}. A nitrogen gas flow of 200 mL·min^{-1} were applied to thermogravimetry chamber

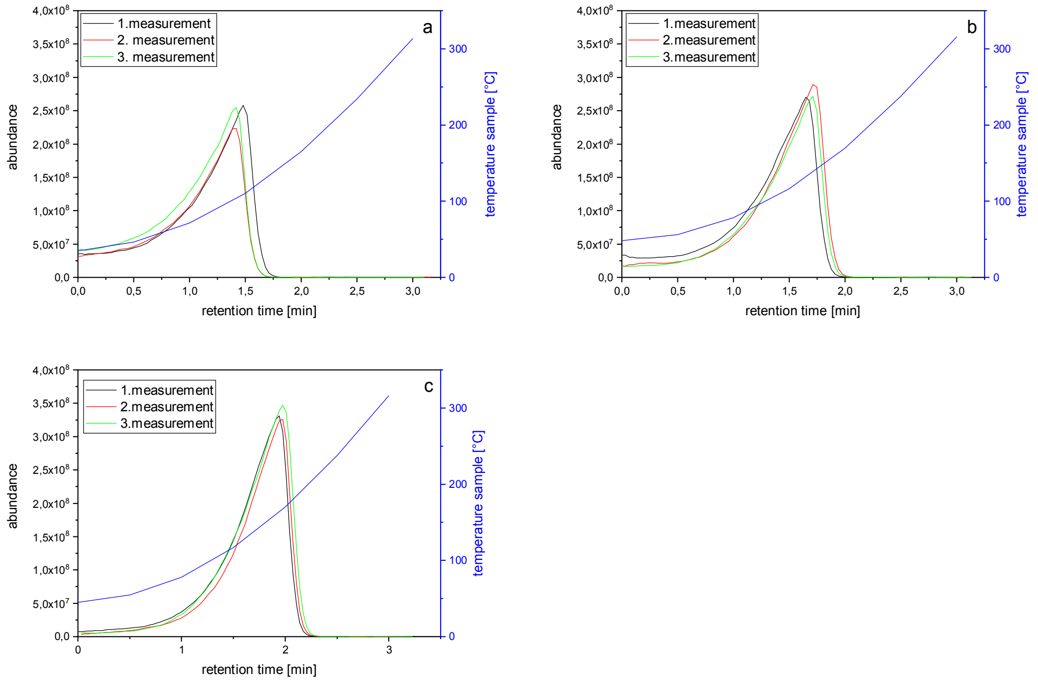

Fig. S2 Total ion chromatograms of single standard analysis using TG-APPI-qMS from a: D4; b: D5; c: D6. For this preliminary study authentic standards of the three cyclic siloxanes were used. In each chromatogram three different measurements with three individual weights for each cyclic siloxane (black = first measurement; red = second measurement; green = third measurement). Additionally, the real temperature gradient in the TG oven is shown in blue. The exact temperature is displayed in blue and on the right y-axis. The standards were analysed as triplicates using a thermogravimetry instrument. About 1 mg of the pure substance was weighted into an alumina crucible and were heated up from 50 °C to 500 °C with a gradient of 150 °C·min^{-1}. 11 mL·min^{-1} of the total 200 mL·min^{-1} nitrogen flow was transferred to the ion source, where the analytes were ionized by a krypton VUV lamp (emits at 117 and 124 nm, respectively). The substances were analysed with a scan range of 50 – 700 Da and a dwell time of 2000 ms

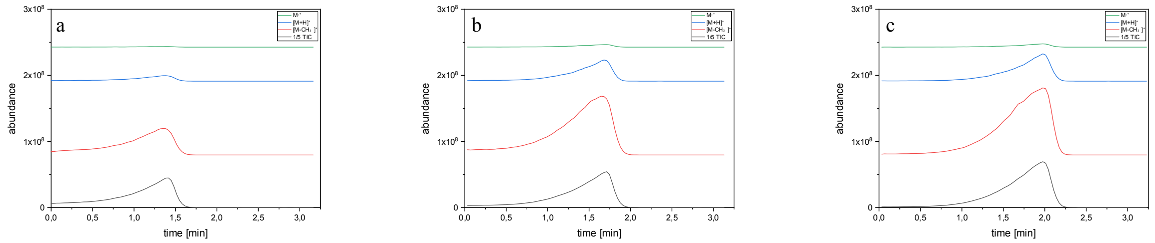

Fig. S3 Comparison of three different, possible adducts of cyclic siloxanes formed in the APPI source during the TG-APPI-qMS analysis. 1/5 of the TIC (black) and the adducts $[M]^{+\cdot}$ (green), $[M+H]^+$ (blue) and $[M-CH_3]^+$ (red) are shown for a) D4 (m/z $[M]^{+\cdot}$ = 296 ± 0.3; $[M+H]^+$ = 297 ± 0.3; $[M-CH_3]^+$ = 281 ± 0.3) b) D5 (m/z $[M]^{+\cdot}$ = 370 ± 0.3; $[M+H]^+$ = 371 ± 0.3; $[M-CH_3]^+$ = 355 ± 0.3) and c) D6 (m/z $[M]^{+\cdot}$ = 444 ± 0.3; $[M+H]^+$ = 445 ± 0.3; $[M-CH_3]^+$ = 429 ± 0.3). For this preliminary study authentic standards of the three cyclic siloxanes were used. The standards were analysed as triplicates using a thermogravimetry instrument. About 1 mg of the pure substance was weighted into an alumina crucible and were heated up from 50 °C to 500 °C with a gradient of 150 °C·min^{-1}. 11 mL·min^{-1} of the total 200 mL·min^{-1} nitrogen flow was transferred to the ion source, where the analytes were ionized by a krypton VUV lamp (emits at 117 and 124 nm, respectively). The substances were analysed with a scan range of 50 – 700 Da and a dwell time of 2000 ms

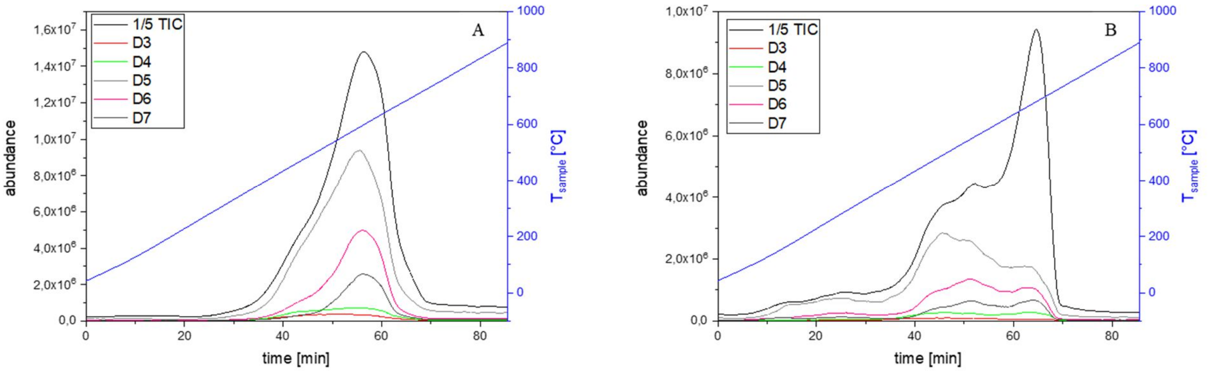

Fig. S4 TG-APPI-qMS data from films of a) manufacturer A and b) manufacturer B. In each chromatogram the TIC is shown in black with one-fifth of the intensity, the EICs of the adduct $[M-CH_3]^+$ from D3 (m/z = 207 ± 0.3; red), D4 (m/z = 281 ± 0.3; green), D5 (m/z = 355 ± 0.3; grey), D6 (m/z = 429 ± 0.3; pink) and D7 (m/z = 503 ± 0.3; dark grey). The right y-axis and the blue line in the diagram display the measured sample temperature during analysis. The films were analysed as triplicates. About 5 mg of the chosen film was weighted into an Al_2O_3 crucible and were heated up from 30 °C to 900 °C with a gradient of 10 °·min^{-1}. 11 mL·min^{-1} of the total 200 mL·min^{-1} nitrogen flow was transferred to the ion source, where the analytes were ionized by a krypton VUV lamp. The substances were analysed with a scan range of 50 – 700 Da and a dwell time of 2000 ms

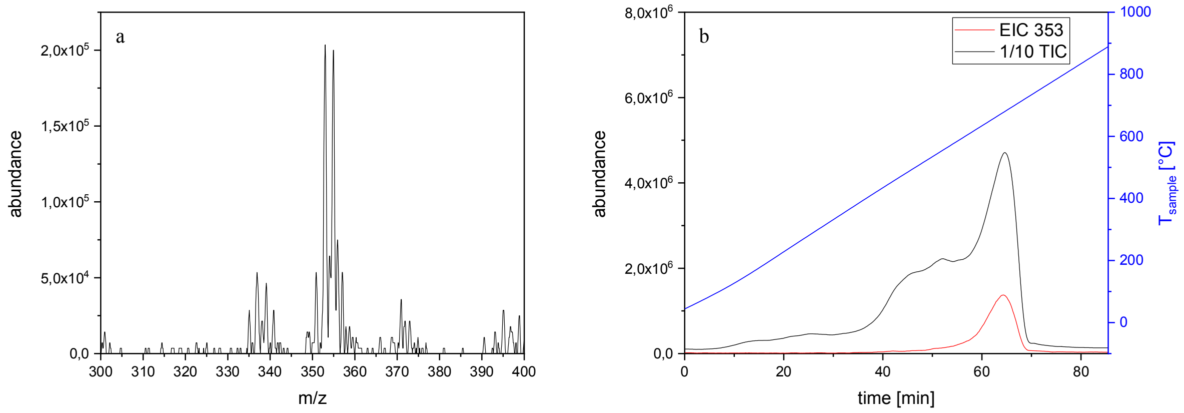

Fig. S5 Further investigations of the PDMS film from manufacturer b. The highest peak of the DTG curve and TIC mainly results from a molecule with an m/z ratio of 353. To illustrate this the mass spectrum at 64.7 min (shown in figure a) and the EIC of 353 ± 0.3 in comparison to the TIC (shown in figure b) are shown. The films were analysed as triplicates using a thermogravimetry instrument coupled to an atmospheric photo ionization quadrupole mass spectrometer. About 5 mg of the chosen film was weighted into an Al_2O_3 crucible and were heated up from 30 °C to 900 °C with a gradient of 10 $°C \cdot min^{-1}$. 11 $mL \cdot min^{-1}$ of the total 200 $mL \cdot min^{-1}$ nitrogen flow was transferred to the ion source, where the analytes were ionized by a krypton VUV lamp (emits at 117 and 124 nm, respectively). The substances were analysed with a scan range of 50 – 700 Da and a dwell time of 2000 ms

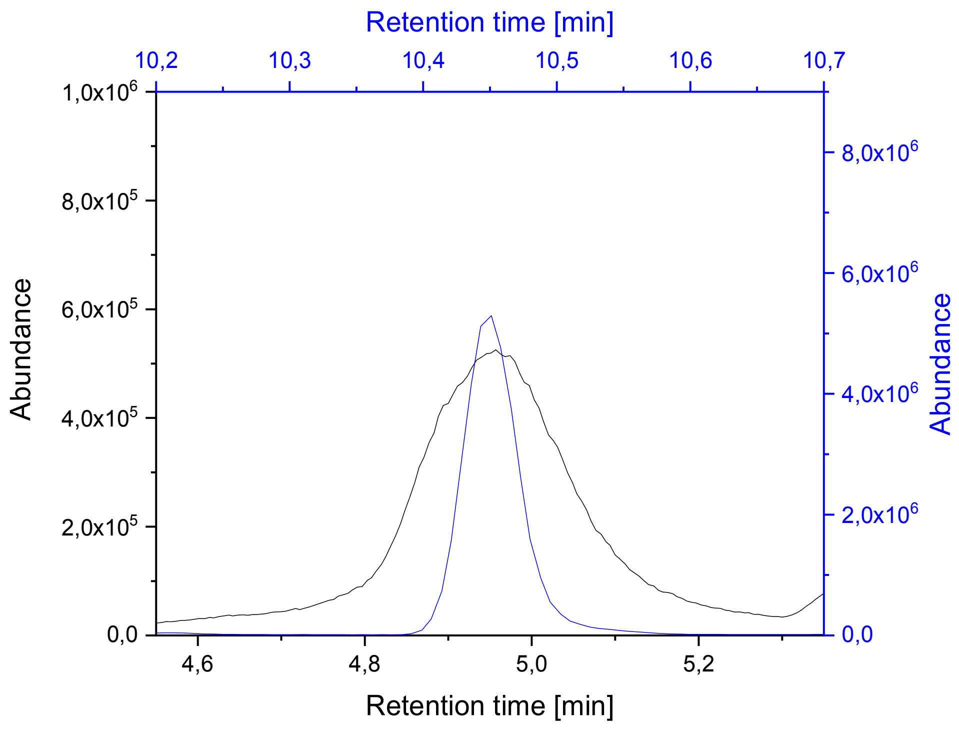

Fig. S6 Illustration of the advantage from the oven cryo cooling device on the example of 2-methylbutanal. Therefore, the multi-standard with a concentration of 50 µM were measured using TFME as a pre-concentration step and the TD-GC-qMS as an analytical instrument. Shown are excerpts from the TICs of a) the measurement without and b) with cryogenic oven cooling. The films were analysed with a thermodesorption gaschromatography mass spectrometer and in the first step thermally desorbed in the TD system at 200 °C using a desorption flow of 60 mL·min^{-1} helium. Subsequently, the desorbed analytes were trapped on a PTV injector at -10 °C. After trapping the analytes the liner was heated up to 270 °C and the substances were analysed with a flow of 1 mL·min^{-1} helium in a gc system with a DB-1 column (30 m x 0.25 mm x 1 µm). The measurement without cryogenic oven cooling have been performed with a temperature gradient from 40 °C to 325 °C with 10 °C·min^{-1}. In contrast to the measurement without cryogenic oven cooling, the measurement were carried out with cryogenic oven cooling with a temperature gradient from -10 °C to 325 °C with 10 °C·min^{-1}. The eluting compounds from both measurements were analysed in a quadrupole mass spectrometer with a scan range of 40 – 600 Da and a scanning rate of 2.24 scans·s^{-1}

Table S1 Comparison of FWHM values of measurements with and without cryogenic oven cooling using the example of six multi-standard metabolites. The measurements were carried out by the methods described below Fig. S6

metabolite	FWHM [s]	
	without	with
2-methylbutanal	11.9	2.7
dimethyl disulphide	9	4.8
2-hexanone	7.2	3
2-heptanone	3.6	2.4
1-octanol	4.2	3
2-nonanone	2.4	1.8
2-aminoacetophenone	3	2.4

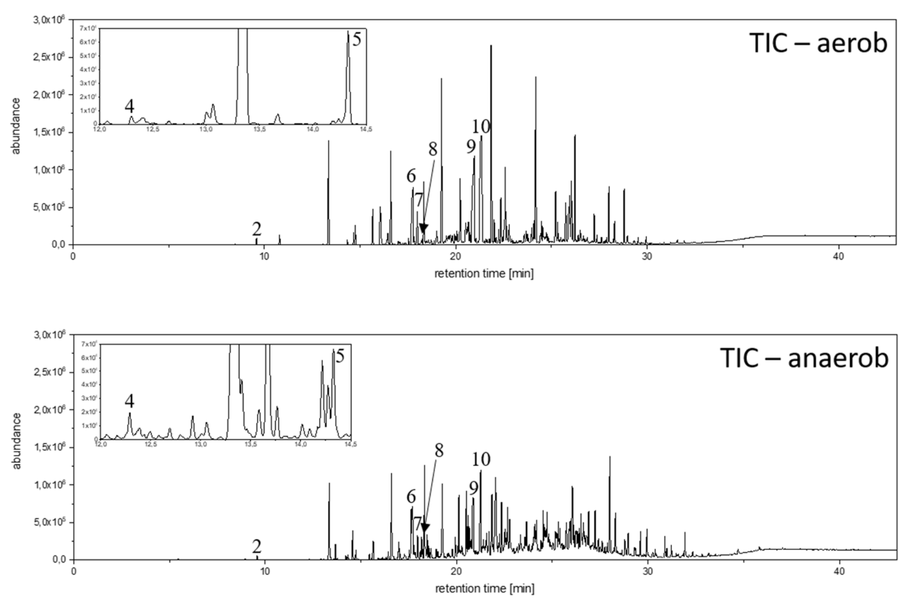

Fig. S7 TICs resulting from TD-GC-qMS measurements. 100 µL of the multi-standard solution, containing 10 possible metabolites of *P. aeruginosa*, was plated onto the nutrient of the biofilm model using aerobic (top chromatogram) and anaerobic conditions (bottom chromatogram). The identified model substances are marked

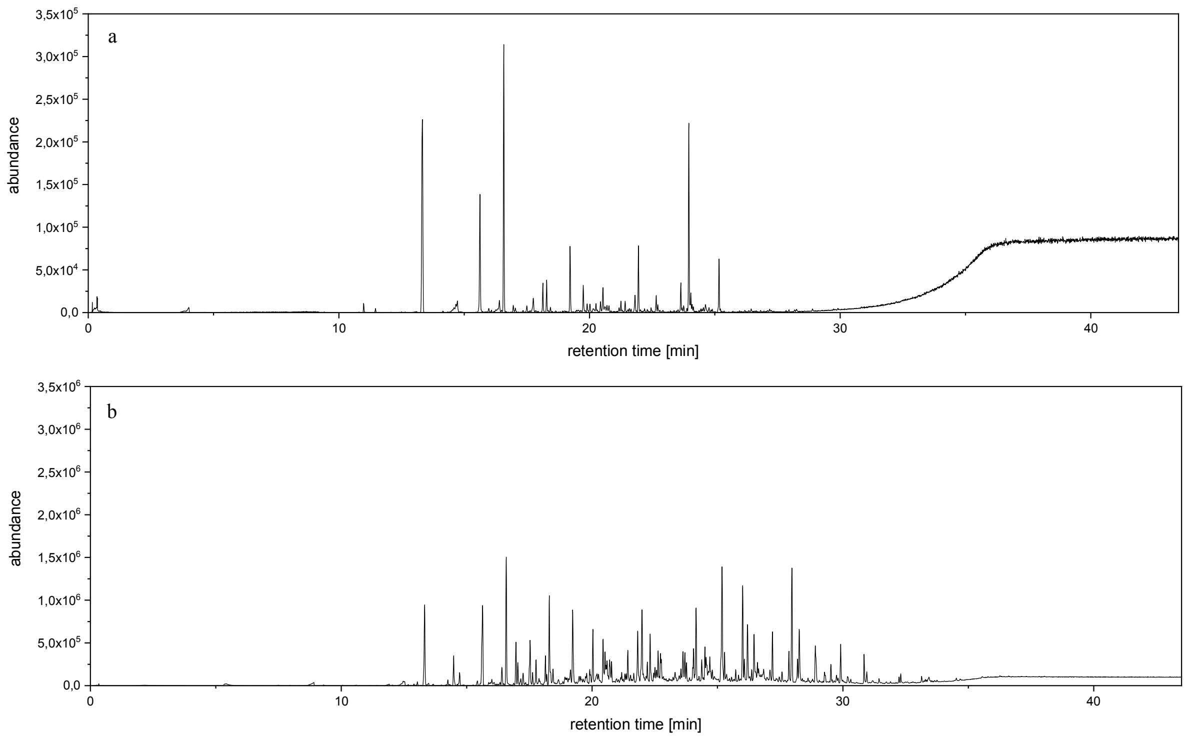

Fig. S8 Procedual blank of the in vitro CF biofilm model. The procedual blank was taken from the in vitro CF biofilm model containing only the nutrient medium. The TIC of the prodecual blank of a) the aerobic culture and b) the anaerobic culture is shown. The films were analysed with a thermodesorption gaschromatography mass spectrometer and in the first step thermally desorbed in the TD system at 200 °C using a desorption flow of 60 mL·min^{-1} helium. Subsequently, the desorbed analytes were trapped on a PTV injector at -10 °C. After trapping the analytes the liner were heated up to 270 °C and the substances were analysed with a flow of 1 mL·min^{-1} helium in a gc system with a DB-1 column (30 m x 0.25 mm x 1 µm). A temperature gradient from -10 °C to 325 °C with 10 °C·min^{-1} was applied. The eluting compounds were analysed in a quadrupole mass spectrometer with a scan range of 40 – 600 Da and a scanning rate of 2.24 scans·s^{-1}

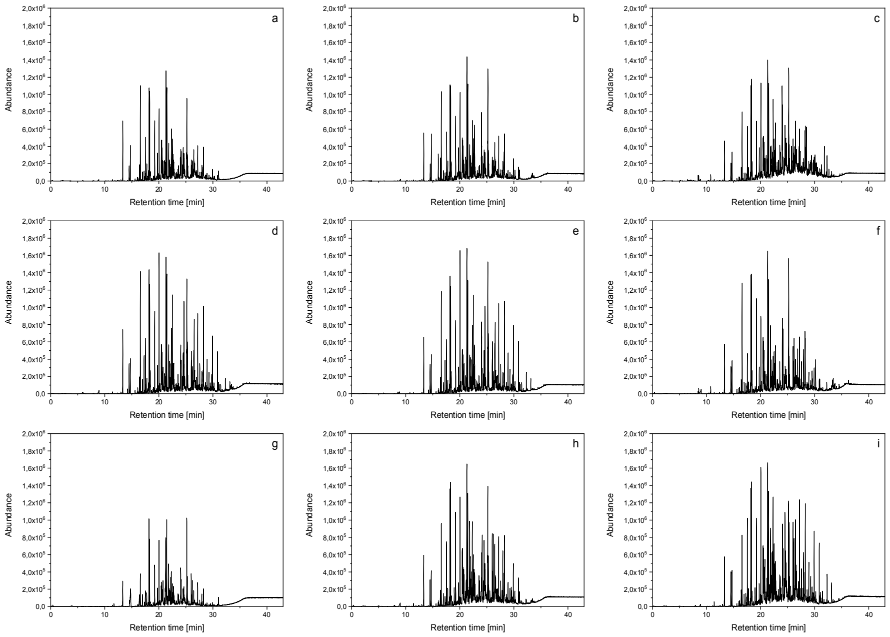

Fig. S9 TICs of the analysis of extracellular volatile metabolites of P. aeruginosa DSM 50071. The biofilms were grown under aerobic conditions. The nine biological replicas are shown from a) first biological replica to i) ninth biological replica

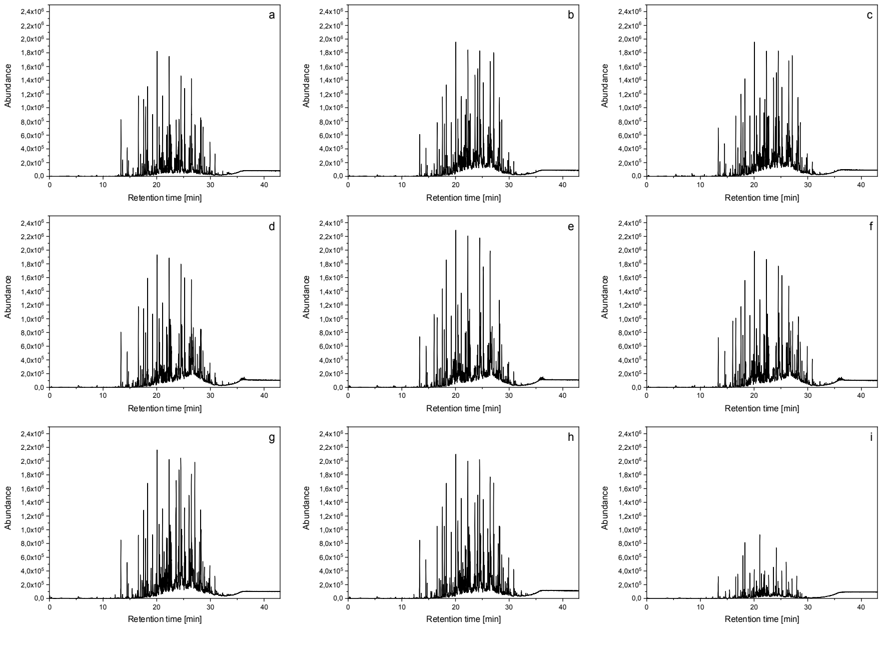

Fig. S10 TICs of the analysis of extracellular volatile metabolites of P. aeruginosa DSM 50071. The biofilms were grown under anaerobic conditions. The nine biological replicas are shown from a) first biological replica to i) ninth biological replica
